# Hyaluronic Acid as an Adjunct to Coronally Advanced Flap Procedures for Gingival Recessions: A Systematic Review and Meta—Analysis of Randomized Clinical Trials

**DOI:** 10.3390/jpm12091539

**Published:** 2022-09-19

**Authors:** Mariana A. Rojas, Lorenzo Marini, Philipp Sahrmann, Andrea Pilloni

**Affiliations:** 1 Section of Periodontics, Department of Oral and Maxillo-Facial Sciences, Sapienza, University of Rome, 00161 Rome, Italy; 2 Department of Periodontology, Endodontology and Cariology, University Centre for Dental Medicine, University of Basel, 4058 Basel, Switzerland

**Keywords:** coronally advanced flap, gingival recession, hyaluronic acid, meta-analysis, randomized clinical trials, systematic review

## Abstract

Previous systematic reviews have reported that coronally advanced flap (CAF) + connective tissue graft (CTG) are the gold standard in root coverage procedures (RCP). Nevertheless, adjunctive treatment with hyaluronic acid (HA) has been proposed to aim at improving clinical outcomes and reducing patient morbidity. The aim of this systematic review and meta-analysis is to compare the use of HA as an adjunctive treatment to CAF procedures in Miller class I and II (recession type 1; RT1) gingival recession (GR) defects treatment with no adjunctive/other treatments. MEDLINE, The Cochrane Central Register of Controlled Trials, Web of Science, Scopus databases and gray literature were searched up to April 2022. The primary outcome variables were mean recession coverage (MRC) and reduction of the recession depth (RecRed). Weighted mean differences and 95% confidence intervals between treatments were estimated using a random-effect mode. From 264 titles identified, 3 RCTs reporting 90 GR defects in 60 patients were included. Overall analysis of MRC and RecRed were 0.27% (*p* = 0.01) and 0.40 mm (*p* = 0.45) in favor of CAF + HA compared to CAF alone/CAF + subepithelial connective tissue graft (SCTG), respectively, with a statistically significant difference only for MRC values. Nevertheless, due to the limited number and heterogeneity of the included studies, well-performed RCTs are needed to clarify a potential advantage of HA in RCPs in the future.

## 1. Introduction

Gingival recession (GR) is characterized by the displacement of the gingival margin below the cemento–enamel junction [1]. It is associated with attachment loss and exposure of root surface to the oral environment and is esthetically unacceptable for many patients when visible [2]. Furthermore, GR is frequently associated with dentin hypersensitivity, cervical caries and non-carious cervical lesions (NCCL) such as abrasions or erosions [2,3]. It affects more than 50% of the population including healthy individuals [4].

Although the etiology of GR has not been totally clarified, several predisposing factors have been reported including thin periodontal biotype [4], absence of attached gingiva [4] and previous orthodontic treatment [5]. Other factors with a low level of evidence were suggested, such as intrasulcular restorative margins in sites with minimal or no gingiva, persistent gingival inflammation, shallow vestibular depth or frenum position that compromises oral hygiene procedures and the presence of tissues deformities (e.g., clefts or fissures) [2,6]. “Improper” toothbrushing has been proposed as the most important etiological factor [3,7]. However, it has been demonstrated that GR can occur in populations with both high and low standards of oral hygiene [8]. Accordingly, a recent systematic review concluded that data available regarding the association between toothbrushing and GR are inconclusive [9].

Aesthetic demand is the primary indication for root coverage surgical procedures (RCPs) [10]. The ultimate goal of RCPs is the resolution of the defect in terms of complete root coverage (CRC), with an esthetic appearance comparable to adjacent healthy soft tissues and minimal probing depth following healing [11,12]. Several surgical procedures have been proposed to treat single and multiple GR defects [13]: (1) pedicle soft-tissue graft procedures: rotational flap [14,15,16] or advanced flap procedures [17,18,19,20,21]; (2) non pedicle soft-tissue procedures: tunnel technique and their modifications [22,23,24]; (3) regenerative procedures with barrier membranes [25] or application of enamel matrix derivative (EMD) [26]; (4) free soft-tissue graft procedures: epithelialized graft [27] or subepithelial connective tissue graft (SCTG) [28]. Nevertheless, although several techniques have been proposed, coronally advanced flap (CAF) + connective tissue graft (CTG) has been accepted as the gold standard in RCPs because of the best outcomes achieved in terms of mean root coverage (MRC), keratinized tissue width (KTW), gingival thickness and aesthetic results [29]. However, some drawbacks as the presence of a second surgical site, limited palatal tissue availability and/or patient morbidity have been described for CTG procedures [30]. Therefore, several CTG substitutes were introduced, including the use of biomaterials (as collagen-based membranes and dermal tissue derivatives) [31] and/or bioactive agents as EMD, which have been associated to successful clinical and histological outcomes when combined with CAF procedure [26]. A recent meta-analysis concluded that, when combined with CAF, CTG is related with an increase in KTW, whereas EMD seems to improve the wound healing process with a reduction in probing depth (PD) values [32].

Regarding the use of bioactive agents, hyaluronic acid (HA) has been extensively used in periodontal therapy in the last years. HA is a major component of the extracellular matrix in almost all body tissues [33], and it is active during the entire process of wound healing, being involved in cell proliferation, migration and tissue remodeling [34,35]. Moreover, it was demonstrated that HA has bacteriostatic, fungostatic, anti-inflammatory, anti-edematous, osteoinductive and pro-angiogenetic properties [34,35]. In the periodontal tissues, it is synthetized by fibroblasts and keratinocytes in the gingiva and by periodontal ligament cells, cementoblasts and osteoblasts [33]. A recent in vitro study has provided evidence on the effects of HA to maintain the viability of oral fibroblasts and increase their proliferative and migratory abilities [36].

HA has been extensively used in non-surgical and surgical periodontal treatment and, based on the outcomes of several studies, it may be suggested that: (1) HA can be considered a promising material for periodontal regenerative/reconstructive surgery [37,38,39,40,41,42]; (2) HA promotes faster wound healing when used in oral soft tissue wounds [43].

Even though the use of HA in periodontal surgical treatment has shown successful short-and long-term clinical results [40,41,42], to date, it is still unclear whether there are any benefits when HA is used as an adjunct to RCPs.

Therefore, the aim of this systematic review and meta-analysis was to evaluate the current evidence regarding the use of HA as adjunctive treatment to CAF procedures in Miller class I and II (RT1; recession type 1) GR defects treatment and compare it with no adjunctive or other treatment modalities.

## 2. Materials and Methods

### 2.1. Protocol and Registration

#### 2.1.1. Reporting Format

This systematic review was developed and structured according to the PRISMA (Preferred Reporting Items for Systematic Review and Meta-Analyses) Statement [44]. Beforehand, the review was registered in PROSPERO (registration number: CRD42022340155).

The focused question was designed using the following PICOS [45] (Population (P), Intervention (I), Comparison (C), Outcomes (O) and Study Design (S)) definitions:

- Population: systemically healthy patients with Miller class I or class II [46] (RT1) [47] GR defects;
- Intervention: CAF + HA;
- Comparisons: control treatment modalities were: (1) CAF alone, (2) CAF + CTG and/or other biomaterials;
- Outcomes: The primary outcome variables were: mean root coverage (MRC) and reduction of the recession depth (RecRed, obtained from the difference between the baseline recession depth and final recession depth). Secondary outcome variables included: complete root coverage (CRC), probing depth (PD), clinical attachment level (CAL), keratinized tissue width (KTW) and patient-reported outcome measures (PROMs, in terms of post-operative morbidity during at least the first post-operative week);
- Study design: the present systematic review was restricted to randomized clinical trials (RCTs).

Accordingly, the focused question was:

In systemically healthy patients with Miller class I or class II (RT1) GR defects (P) undergoing surgical interventions with a CAF procedure, does HA (I) provide any advantage when compared to no or other adjunctive treatment modalities (C) in terms of postoperative MRC and RecRed or other clinical outcomes (O)?

#### 2.1.2. Eligibility Criteria

The studies were selected according to the following criteria:

Inclusion criteria

- Randomized clinical trials (RCTs);
- Studies comparing CAF + HA with CAF alone, CAF + CTG and/or in combination with other biomaterials (CAF + biomaterial) in patients with Miller class I or class II (RT1) GR defects;
- Information regarding specific properties of the HA used (type, concentration and application method);
- Follow-up period ≥ 6 months;
- Otherwise periodontally and systemically healthy patients.

Exclusion criteria

- In vitro and animal studies;
- Retrospective studies;
- Case series, case reports and reviews;
- Presence of systemic disease or active periodontal disease.

### 2.2. Search Strategy

#### 2.2.1. Electronic Search

A comprehensive and systematic electronic search of US National Library of Medicine MEDLINE (Pubmed), The Cochrane Central Register of Controlled Trials (CENTRAL), Web of Science and Scopus databases until April 2022 was performed. No restrictions on publication time or language were applied [48].

Grey literature search was conducted through the registering of clinical studies hosted by the US National Institutes of Health, the Literature Report (www.nyam.org/library/collections-and-resources/grey-literature-report/ (accessed on 30 April 2022)) and the OpenGrey databases (www.opengrey.eu) (accessed on 30 April 2022).

The strategy used was a combination of the following MeSH (Medical Subject Headings) terms: (Gingival recession OR Root coverage OR Periodontal plastic surgery OR Gingival surgery OR Mucogingival surgery OR Cosmetic periodontal plastic surgery OR Coronally advanced flap) AND (Hyaluronic Acid OR Hyaluronan).

#### 2.2.2. Manual Search

A manual search was performed in the table of contents of the following journals considered relevant primary sources related to the topic: *Clinical Oral Investigations, Journal of Periodontology, Journal of Clinical Periodontology, Journal of Periodontal Research, and International Journal of Periodontics & Restorative Dentistry, Journal of Dentistry, and Journal of Indian Society of Periodontology,* for articles that were published until April 2022 without restrictions on dates. In addition, the reference list of included studies and systematic reviews on hyaluronic acid were assessed to capture any possible additional records, as suggested by Greenhalgh and Peacock [49].

### 2.3. Study Selection Process

Previous to the screening process, the first 50 titles and abstracts retrieved by the electronic literature search were used to calibrate the two reviewers (LM and MR) with a senior researcher (AP). Inter-reviewer agreement was calculated as kappa coefficient. Consequently, titles and abstracts were independently screened by two reviewers (LM and MR). From studies potentially meeting the inclusion criteria, full texts were obtained and assessed for possible inclusion. Disagreement between the reviewers was resolved by discussion with a third reviewer (AP).

### 2.4. Data Extraction

Studies fulfilling the eligibility criteria were processed for data extraction by two reviewers (LM and MR). Data extraction was performed for the following issues:

- Author/title/year of study, study affiliation data;
- Study design and follow-up period;
- Sequence generation;
- Allocation concealment;
- Blinding of participants and outcomes assessors;
- Population characteristics;
- Pretreatment;
- Intervention site characteristics, number and localization of GR defects treated;
- Surgical technique;
- HA-related information;
- Control treatment characteristics;
- Post-interventional medication;
- Post-surgical instructions;
- Maintenance therapy;
- Primary and secondary outcomes;
- Information on study funding.

In case of missing or unclear data the authors of the respective studies were contacted via email.

### 2.5. Quality Assessment of Included Studies (Risk of Bias)

The quality assessment of the included studies was performed independently by LM and MR following the guidelines of the Cochrane Collaboration [50] and using the Risk of Bias 2.0 tool, (RoB 2) [51].

Each study was analyzed considering six domains: (1) sequence generation, (2) allocation concealment, (3) blinding of participants and outcome assessors, (4) incomplete outcome data, (5) selective outcome reporting and (6) other sources of bias. In each assessment tool previously mentioned, a judgement of “Yes” or “No” indicated low and high risk of bias, respectively, whereas “Unclear” judgement indicated uncertain risk of bias.

A study was assigned as “Low risk of bias” when all the domains were of low risk of bias. However, when one or more key domains resulted with unclear or high risk of bias, the study was assigned as “Unclear or High risk of bias”.

Disagreements on data extraction and quality assessment were discussed and resolved by consensus. A third reviewer (AP) was consulted when necessary.

### 2.6. Data Analysis and Heterogeneity Assessment

Meta-analyses were conducted, and forest plots were calculated based on data from studies reporting comparable treatment and outcomes.

The continuous variables (MRC and RecRed) were analyzed using Review Manager 5.4 (Review Manager, RevMan, Version 5.4, The Cochrane Collaboration, Copenhagen, Denmark, 2020).

Random effects model was implemented. The estimates of the intervention effects (mean difference) were expressed as percentages or millimeters with 95% confidence intervals (CIs). Heterogeneity was assessed using a chi^2^ test and the I^2^ statistic.

The statistical level of significance of the effect of meta-analysis was set at *p* ≤ 0.05.

## 3. Results

### 3.1. Search and Screening

The electronic search identified 264 titles (91 from MEDLINE/Pubmed, 29 from the Cochrane Central Register of Controlled Trials, 65 form the Web of Science and 79 from Scopus).

**Figure 1:**
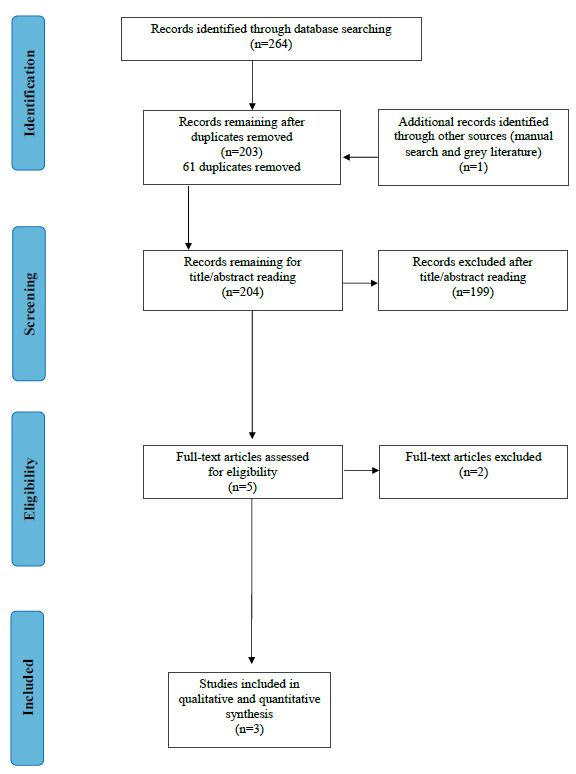
Flow diagram (PRISMA format) of the screening and selection process.

After duplicates removal, 203 records were screened for title/abstract reading. One additional record was retrieved from manual search. No additional articles were identified through grey literature. Accordingly, 204 records were available for title and abstract assessment. In the first step, 199 articles were excluded. Of the remaining five full-text publications two were excluded due to: report data of a previous study [52]; type of the study: meeting abstract [53]. Three articles [41,54,55] remained for qualitative and quantitative analysis (Figure 1).

Calibration among authors indicated high agreement for title and abstract screening (k-score = 0.92, agreement = 92%) and complete agreement (k-score = 1, agreement = 100%) for the full text screening.

### 3.2. Description of the Included Studies

#### 3.2.1. Study Design

The characteristics of the included studies are reported in Table 1. Studies were conducted between 2014 and 2019. Regarding the type of the study, one was a parallel (double-arm) RCT [41], while the other two studies were designed as a split-mouth RCT [54,55]. Follow-up periods were reported at 6 [55], 9 [54] and 18 [41] months.

Pilloni and co-workers’ RCT [41] was a single-blinded study, whereas in the other two studies [54,55] this data was not reported. In two of the included studies [41,55], the test groups (CAF + HA) were compared with CAF alone (control group), while in the other study [54], the control group was CAF + SCTG.

Power calculation was performed in two studies [41,55]; however, data concerning the sample size calculation in one of the studies [55] was not clear.

All the studies were conducted in university settings.

No financial support was provided by any company for any of the studies.

#### 3.2.2. Population Characteristics

A total of 60 patients (50% males and 50% females) with an age range between 21 and 47 years were assessed in the included studies. All studies reported gender distribution while only two studies reported the age of the patients [41,54]. Smokers were excluded in all the studies.

Data on 90 Miller class I or class II (RT1) GR defects (45 test and 45 control: 25 CAF alone and 20 CAF + SCTG) were presented. The localization of the treated defects was specified in one of the studies [41]. In the remaining studies [54,55], this information was restricted on the inclusion criteria (upper or lower central incisors, lateral incisors, canines and first premolars sites: two Miller class I/II GR defects per patient [54] or canine and premolar sites: two Miller class I GR defects per patient [55]).

Teeth with abrasion of the cemento–enamel junction (CEJ) were excluded [55] or previously treated with composite to reconstruct the CEJ before surgery [41]. In the remaining study [54], the respective information was not available, but the authors excluded teeth with cervical restorations [55]. Patient’s and teeth and defect characteristics at baseline are presented in Table 2.

#### 3.2.3. Treatment Characteristics (Intervention/Comparison)

- Pre-treatment

Modifications of oral hygiene habits were described in two of the studies [41,54]. In one of the studies [41] scaling and root planning was performed two months before surgery, while in the remaining studies, this information was not available [54,55].

- Surgical procedure

The surgical procedure was similar in all the included studies (CAF for single GR treatment by Zucchelli et al. [56]). The main difference was found in the design of the incisions/flap (trapezoidal [54,55] versus triangular design [41]).

The root surface treatment was different in all the studies: after flap elevation, gentle root planning was performed using a curette up to 1 mm from bone crest [41] or the root surfaces were planned before the elevation of the flap, and the anatomical landmarks were not specified [55]. In one of the studies [54], this information was not reported.

The specific treatment in the test group differs in the HA used (a gel of 1.6% cross-linked HA + 0.2% natural HA [41] versus 0.2% HA gel [54,55]). In both studies, HA was applied on the root surface before flap coronal displacement and suture (with a sterile instrument [54,55] or with a syringe specific for its application [41]).

In all the studies non-resorbable suture materials were used (4-0, [54] 5-0, [55] and 6-0 [41] sutures). Regarding the material, monofilament nylon and polypropylene sutures were used in the most recent performed study [41], while this information was not reported in the other two studies [54,55].

- Post-surgical medication and maintenance

In the most recent study [41], patients received ibuprofen 600 mg at the end of the surgical procedure and were instructed to take another tablet 6 h later (with subsequent doses only if needed). Moreover, Amoxicillin (1 g every 12 h) was provided during 5 post-surgical days. Kumar and co-workers [55] recommended ibuprofen + paracetamol (three times daily, TID) in case of pain. In this study, antibiotic was not prescribed, while in the remaining study [54], analgesic and antibiotics were prescribed to all the patients, but the authors did not specify the administration protocol.

Post-surgical chlorhexidine (CHX) rinses (60 s) were indicated in all the studies, but differences in the concentration and duration were observed, which ranged from 0.12% CHX for 15 days [41], versus 0.2% CHX for ten days [55] or three weeks [54].

The suture removal was performed after 7 days, and patients were instructed to clean the surgical sites with a cotton pellet soaked in a 0.2% CHX for the following 10 days [55], after 10–14 days [54] or after 14 days [41]. Patients were instructed to brush with a post-surgical soft toothbrush after two [41] or three [54,55] weeks.

The maintenance protocol was not described in two of the studies [54,55]. In the study by Kumar and co-workers [55], patients were recalled after 1, 3, 6, 12 and 24 weeks. On each visit, the site was checked for meticulous plaque control without subgingival instrumentation until the 6th week.

In the other study [41], the patients were recalled for professional oral hygiene, maintenance procedures and clinical measurements as needed: after 1, 2 and 4 weeks and after 3, 6, 12, 15 and 18 months post-surgery. The use of a soft toothbrush was discontinued only after 3-month follow-up. The information concerning the period necessary to resume oral hygiene procedures is absent in two of the studies [54,55].

### 3.3. Primary and Secondary Parameters

RD, MRC, PD and CAL were evaluated in all the studies while KTW was evaluated in two of the studies [41,54]. One study also evaluated the gingival index (GI) and plaque index (PI) [54]. CRC was assessed only in one study at 18 months follow-up [41].

The primary outcome in the parallel designed RCT [41] was RecRed, whereas this was not clear for the other studies [54,55].

In the study performed by Pilloni and co-workers [41], post-operative patient morbidity (pain, swelling and discomfort) was assessed as secondary parameter using a visual analogue scale (VAS) questionnaire after 1 week. In the remaining studies [54,55] this evaluation was not performed.

In all the included studies [41,54,55], the clinical parameters were obtained using a UNC 15 probe. In two studies [54,55], a stent of acrylic was used that allowed a reproducible periodontal probe position to record the measurements pre- and post-surgically. Clinical parameters were measured at baseline and after 18 months for the most recent study [41]. In the other two studies, clinical measurements were performed at different time points: after 1, 3 and 9 post-surgical months for the Rajan and co-workers study [54], and after 1, 3, 6, 12 and 24 post-surgical weeks for the remaining study [55].

#### 3.3.1. Primary Outcome Variables

- MRC and RecRed

Both treatments resulted in considerable advantages regarding the primary outcomes variables.

Meta-analysis was performed for MRC and RecRed with data collected from the three included studies [41,54,55], and showed statistically significant benefit of HA use as an adjunctive treatment to CAF in terms of MRC (MRC: 0.27%; *p* = 0.01; CI 95%—0.15; 0.70) in comparison to CAF alone/CAF + SCTG. However, no statistically significant difference was observed for RecRed values (0.40 mm; *p* = 0.45; CI 95%—0.02; 0.82).

Statistical heterogeneity was high in MRC (I^2^ = 76%) while was absent in RecRed (I^2^ = 0%).

Secondary outcome variables, likewise, as well as MRC and RecRed values at further time points [54,55], could not be evaluated in a meta-analysis due to methodological heterogeneity.

**Figure 2:**
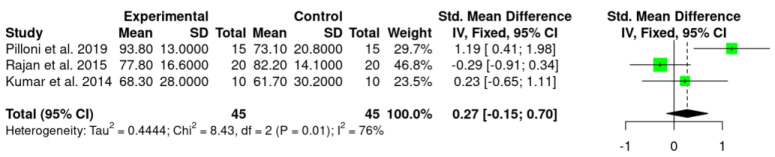
Forest plot with respect to the primary outcome (MRC).

**Figure 3:**
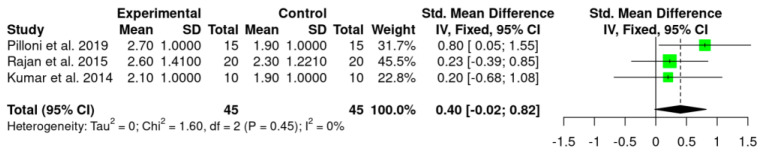
Forest plot with respect to the primary outcome (RecRed).

Details regarding the primary outcomes (i.e., MRC and RecRed) are presented in Figure 2 and Figure 3, respectively.

The percentage of root coverage ranged from 58.4 ± 8.8% to 93.8 ± 13.0% for the test group and 48.1 ± 13.4% to 73.1 ± 20.8% for control group. Although all studies reported significant changes in MRC values in both groups, when compared to the baseline values, only one study [41] reported higher values for the test group, showing a statistically significant difference (MRC: 93.8 ± 13.0% versus 73.1 ± 20.8% for test and control groups, respectively). In one study [54], inter-group comparison showed moderately significant difference between groups at 3 months, with a higher % of root coverage for HA group (58.4 ± 8.8%) versus 48.1 ± 13.4 % for the control group. However, at 9 months this difference was not observed.

Regarding RecRed, only in the study [41] was this parameter calculated, showing higher values in the test group than in the control group (2.7 mm [1.0] versus 1.9 mm [1.0]), and the difference was statistically significant. In the other two studies [54,55], recession depth (RD) changes were presented. Rajan and co-workers [54], showed moderately significant difference in RD between groups at 1 month (2.05 ± 0.69 for test group versus 2.45 ± 1.05 for control group). Nevertheless, no significant difference was observed when inter-group comparison was performed at 3 and 9 months.

In the study by Kumar and co-workers [55], no inter-group difference was found for the different time points assessed (i.e., 1, 3, 6 12 and 24 weeks). After 24 weeks, mean RD was 1.1 mm ± 0.99 mm and 1.0 mm ± 0.66 mm for test and control group, respectively. Based on the values presented by the authors in the previous studies [54,55], we have calculated the RecRed, and added this information in Table 3. In both studies, no significant differences were observed at the final evaluation between test and control group (2.6 ± 1.09 versus 2.3 ± 0.94 [54] and 2.1 ± 0.99 versus 1.9 ± 0.73 [55], respectively).

Detailed information regarding primary outcome variables is summarized in Table 3.

#### 3.3.2. Secondary Outcome Variables

- Complete root coverage (CRC)

CRC was evaluated only by the Pilloni and co-workers study [41]. The authors reported a significant difference between groups. CRC was obtained in 80% of the GR defects treated in the HA group while in the control group CRC was observed in 33.3% of the GRs defects treated.

- Probing depth (PD)

Two studies [41,55] reported no significant difference in PD values when inter-group evaluation was performed. In the Pilloni and co-workers’ study [41], PD was found to be slightly, but statistically significantly, increased in both groups (baseline value for control and test group: 1.0 [0.0] versus final values: 1.0 [1.0] for the HA and 2.0 [1.0] for the control group). Instead, in the Rajan and co-workers study [54], the mean PD was reduced significantly in both groups 9 months post therapy (HA group: baseline value 2.79 ± 0.63 versus final value 1.15 ± 0.75; control group: baseline value 2.30 ± 0.47 versus final value 0.50 ± 0.51). Inter-group comparison also showed significant difference at 3 months (1.60 ± 0.68 for the HA group versus 1.10 ± 0.31 for the control group) and at 9 months (1.15 ± 0.75 for the HA group versus 0.50 ± 0.51 for the control group). In the remaining study [55], no significant intra- or inter-group changes between baseline and final evaluation were observed.

- Clinical attachment level (CAL)

Significant CAL-gain was observed for both groups in all the studies. Inter-group comparison showed significant differences in two of the studies [41,54]. Nevertheless, the CAL-gain value was calculated only in one study [41]. Therefore, we have calculated this parameter. Pilloni and co-workers [41] observed higher CAL-gain for the test group after 18 months (3.0 [1.0] for the HA group versus 2.0 [1.0] for the control group). In the Rajan and co-workers study [54], higher CAL-gain for the test group was observed at 3 months (3.55 ± 1.10 versus 2.85 ± 0.85) whereas at the final examination (9 months) CAL-gain value was higher in the control group (4.8 ± 0.91 versus 4.2 ± 0.91).

- Keratinized tissue width (KTW)

Two studies evaluated KTW [41,54]. Rajan and co-workers [54] observed an increase in KTW when the baseline and final (9 months) values were compared. Significant difference between groups was reported only at baseline (2.00 ± 0.65 for control group and 2.50 ± 0.61 for test group).

In the remaining study [41], the KTW value did not change for both groups when the baseline and 18 months values were compared. In both studies no significant differences were observed when inter-group comparison was performed. When KTW gain was calculated, in one of the studies the control group showed slightly higher values than test group (1.3 ± 0.73 versus 0.7 ± 0.95) [54]. In the third study, however, no gain in KTW was observed (0.0 [0.0] versus 0.0 [1.0] for control and test group, respectively) [41].

- Patient-related outcome measures (PROMs)

Only one study evaluated post-operative morbidity (pain intensity, discomfort and swelling) after 7 post-surgical days using a VAS [41]. The authors reported that, whereas swelling and discomfort were statistically significantly lower in the test group, no difference was found regarding pain intensity.

In the study by Kumar and co-workers [55], adverse effects such as inflammation, bleeding on probing (BoP), pain and abscess formation were assessed post-surgically. The authors reported that no adverse events were recorded during the post-operative period. Nevertheless, they did not clarify how and when this evaluation was performed.

### 3.4. Risk of Bias Assessment

Detailed risk of bias assessment is demonstrated in Table 4. Two studies [54,55] demonstrated an unclear risk of bias while one study [41] showed a high risk of bias.

## 4. Discussion

The present systematic review assessed the effect of HA as adjunctive treatment to CAF procedure in Miller class I and II (RT1) GR defects surgical treatment. To the best of the authors knowledge, this is the first systematic review and meta-analysis of RCTs investigating the use of HA in RCPs. The results of the meta-analysis showed statistically significant differences regarding MRC (0.27%) in favor of CAF + HA compared to CAF alone/CAF + SCTG (*p* = 0.01, Figure 2). Nevertheless, this finding should be considered with caution as the difference is too small and might not be clinically relevant. In addition, these results included either CAF and CAF + SCGT in the control group, which represent an important limitation, since in the Rajan and co-workers study [54], the control group (CAF + SCTG) showed higher MRC values compared to the HA group (although the difference was not statistically significant). Instead, if we compared the two studies that used CAF alone as control group [41,55], both showed higher MRC values for HA group. For RecRed, although a trend in favor of HA (0.40 mm) was revealed, the difference was not statistically significant (*p* = 0.45, Figure 3) and, regardless of the control group (SCTG or CAF alone), HA group showed higher RecRed values. It is important to highlight that when the values of the control groups of the three included studies were compared, CAF + SCTG showed higher values than CAF alone for both RecRed and MRC parameters.

Although CAF + CTG does still represent the gold standard in RCPs [29], adjunctive treatment options, have gained a place in recent years [30]. Regarding this, the following points could be highlighted of the most recent systematic reviews: (1) although the use of a CTG, with and without the application of EMD, achieved similar CRC and MRC values, the adjunctive treatment with EMD may improve RecRed and CAL-gain values [57]; (2) the use of recombinant human platelet-derived growth factor (rhPDGF) produces no significant regenerative advantage in terms of GR defects treatment [58]; (3) the adjunctive use of platelet-rich fibrin (PRF) improved the percentage of relative root coverage (rRC) and CAL values compared with CAF alone in cases with adequate baseline KTW. However, when the baseline KTW is limited, the use of CTG may be preferred over PRF [59]; (4) no statistically significant differences have been reported for rRC, PD, CAL and KTW when PRF or EMD were used as an adjunct to CAF procedures for Miller class I and II GR defects treatment [59].

A very recent systematic review aiming to assess the efficacy of CAF + CTG compared to alternative approaches for the treatment of single GR defects (i.e., CAF + acellular dermal matrix grafts -ADMG, CAF +EMD and CAF+ xenogeneic porcine collagen matrices -XCM and PRF) concluded that CAF + CTG must be considered the gold standard for the treatment of single GR defects [60]. Nevertheless, as previously mentioned, there is a need for alternatives that can improve the clinical outcomes, and also the post-treatment quality of life since, it has been observed that the incidence of adverse effects in RCPs (mainly discomfort and/or pain) are directly related to the donor sites [60,61]. For this reason, decision making strictly based on scientific evidence is needed.

Hyaluronan has shown promise in the field of regenerative therapy. In fact, the application of HA in periodontal regenerative surgery has been recently evaluated through an animal study and the results showed histological evidence of root cementum, periodontal ligament, and bone formation, suggesting that the clinical improvements reported following the use of this material may indeed reflect periodontal regeneration [62]. Furthermore, it promotes wound healing by its angiogenic properties, increasing cell migration and proliferation and improving tissue hydration [34,35]. This could contribute to a predictable and a better stability of the root coverage obtained. Wound stability maintenance is a key factor in achieving successful outcomes in periodontal regenerative procedures, and it has been demonstrated that post-surgical topical application of HA reduces the wound healing time [43]. Shorting this critical time-period might also help to improve the wound stability. The stability of the wound achieved at the surgical sites when HA is used might have mainly contributed with root coverage.

Irrespective of the histological and clinical healing features, HA has demonstrated several beneficial effects related mainly with the early phases of wound healing process [33,34,35,63,64,65]. All these characteristics could explain the optimal short-term clinical response (after 1 and 3 post-surgical months) reported by one of the included studies [54]. Regarding this, it is important to emphasize that the effect of time on the stability of post-surgical results for RCPs was reported as an important factor [66]. Although 6 months has been considered as a sufficient time for healing and tissue stability [4], it has been reported that the tissue in completely mature after 12 months [67]. It has also been reported that CTG-based techniques show the least changes over time [64,66]. In the present systematic review, only one of the included studies presented a follow-up greater than 12 months (Pilloni and co-workers [41], 18 months follow-up); whereas, in remaining RCTs the final examination was performed at 9 [54] and 6 [55] months. Taking into account the above-mentioned, the different follow-up periods present a limitation, and have to be considered when comparing the results of the single studies.

The qualitative analysis also demonstrated that, regarding the primary outcomes, significant higher MRC and RecRed in favor to the HA group were observed only in the Pilloni and co-workers study [41]. However, the double-arm design of the study could be an important bias since many intra-individual patient-related factors can influence the results. In fact, this could explain, in part, the great difference observed in CRC values obtained in this study (80% test group versus 33.3% control group). Nevertheless, it is interesting to note that, MRC and CRC values obtained in the test group (93.8% and 80%, respectively) are very similar to those obtained with CAF + SCTG (MRC 93.8% and CRC 79%) in another clinical study [68].

The effect of HA in PD-reduction and CAL-gain after non-surgical periodontal treatment [40] and surgical infra-bony defects treatment [42,43] has been previously reported. Among the studies included, only Rajan and co-workers [54] found a significant difference in PD value, with lower value in the test group at 3 and 9 post-surgical months. However, it is important to pointed out that PD-reduction was not analyzed, and the statistically significant difference might not be present if the difference between baseline and final values were calculated. Significant higher CAL-gain for the HA group was observed in two of the studies [41,54], although in Rajan and co-workers [54] study this difference was present after 3 months whereas at the final evaluation CAL-gain value was higher in the control group.

Regarding KTW, a meta-analysis has concluded that when combined with CAF, CTG contributed more to the KTW increase [32]. In fact, when KTW-gain was calculated in the Rajan and co-workers study [54], a higher value in the control group (CAF + SCTG: 1.3 ±0.73 versus test group: 0.7 ±0.95) was observed, even when the baseline values were significantly higher in HA group (2.50 ± 0.61) than in control group (2.00 ± 0.65).

Some weaknesses of this study should be highlighted. First, the few studies available in the literature and, consequently, the number of the included studies. Nevertheless, the small number of studies allows a clear determination of the different approaches performed, and the evidence on HA use might be better reflected in the current form. In our search, we found three articles that met the inclusion criteria: two of those studies [54,55] presented an unclear risk of bias while the other one [41] showed a high risk of bias, which increases the inconsistency of the results. Pilloni and co-workers study [41] presents a low risk of bias for all the domains evaluated, but we considered “other fonts of bias” since the design of the study was not split mouth. In fact, the authors mentioned this in the discussion as a limitation of the study.

Another important concern to consider regarding the assessed studies is, as mentioned above, that the control group in one of the studies [54] consists in CAF + SCGT and not in CAF alone. This can lead to a misleading interpretation of the meta-analysis results.

Moreover, due to the heterogeneity of the included RCTs, the following relevant data are missing in some of the studies: CRC, RecRed, CAL-gain, KTW-gain and PROMs. In fact, to perform the meta-analysis, in two of the included studies [54,55], RecRed values were calculated through the baseline and final RD values reported by the authors.

Finally, it has been reported that differences in HA specific features (as concentration, molecular weight and linear/cross-linked) modified the therapeutic effect of HA-based preparations [69], and this could account for differences in the results obtained, making their interpretation difficult.

HA could offer several advantages in RCPs including the elimination of a second surgical site, reduction in operating time and increased acceptance of the procedure by the patients. However, literature remains scarce to confirm this. Therefore, future research that addresses the following points should be carried out: (1) compare CAF-HA with: CAF alone and other agents such as EMD and CAF + SCTG, (2) evaluate esthetic- and patient-related outcomes, (3) evaluate long-term clinical parameters reporting also intermediate values and (4) evaluate if the beneficial effect obtained with HA in RCPs is predictable for long time periods (5–10 years).

## 5. Conclusions

HA seems to have a facilitating role in the wound healing process and in the regeneration of periodontal tissues, as evidenced by the literature available. The adjunctive use of HA might have a beneficial effect in CAF procedures. Nevertheless, due to the very limited literature available and the heterogeneity of the included studies, well-performed RCTs are needed to clarify a potential advantage of HA in RCPs in the future.

## Figures and Tables

**Table 1:** Characteristics of included studies.

Author Year	Study Design	Follow-Up	Masking	Intervention (C versus T Group)	Power Calculation	Setting	Funding
Pilloni et al. 2019 [41]	RCT Double-arm	18 mo	Single- blind	CAF versus CAF + HA	Yes	U	No
Rajan et al. 2015 [54]	RCT Split-mouth	9 mo	NA	CAF + SCTG versus CAF + HA	No	U	No
Kumar et al. 2014 [55]	RCT Split-mouth	6 mo	NA	CAF versus CAF + HA	Unclear	U	No

C—control group; CAF—coronally advanced flap; HA—hyaluronic acid; mo—months; NA—not available; RCT—randomized clinical trial; SCTG—subepithelial connective tissue graft; T—test group; U—university.

**Table 2:** Population characteristics.

Author Year	Patient’s Characteristics					Teeth and Defect Characteristics	
	Group	Number of Patients	Gender (m/f)	Years M [IQR] Range	Drop-Out	Number/ Type of Tooth	Number/ Type of GR Defect
Pilloni et al. 2019 [41]	C T	15 15	8/7 8/7	30 [12] 30 [15]	0 0	15 (5 UC, 1 LC, 7 UPM, 2 LPM) 15 (2 UC, 2 LC, 7 UPM, 4 LPM)	15 Miller Class I (RT1) 15 Miller Class I (RT1)
Rajan et al. 2015 [54]	C/T	20	7/13	26–42 years	0	40 NA	40 Miller Class I/II(RT1)
Kumar et al. 2014 [55]	C/T	10	7/3	NA	0	20 NA	20 Miller Class I (RT1)

C—control group; f—female; IQR—interquartile range; LC—lower canine; LPM—lower premolar; m—male; M—median; NA—not available; RT—recession type: T—test group; UC—upper canine; UPM—upper premolar.

**Table 3:** Primary outcome variables.

Author Year		Primary Outcome Variables					
		MRC (%)		RD (M [IQR]/Mean ± SD)		RecRed (M [IQR]/Mean ± SD)	
	Time points	C	T	C	T	C	T
Pilloni et al. 2019 [41]	Baseline	-	-	3.0 [1.0]	3.0 [1.0]	-	-
	18 mo	73.1 ± 20.8%	93.8 ± 13.0% *	0.0 [0.0]	1.0 [1.0] *	1.9 [1.0]	2.7 [1.0] *
Rajan et al. 2015 [54]	Baseline	-	-	3.45 ± 0.94	3.65 ± 1.09	-	-
	1 mo	-	-	2.45 ± 1.05	2.05 ± 0.69 *	-	-
	3 mo	48.07 ± 13.35%	58.43 ± 8.80% *	1.80 ± 0.77	1.50 ± 0.51	-	-
	9 mo	82.15 ± 14.05%	77.84 ± 16.56%	1.15 ± 0.59	1.05 ± 0.76	2.3 ± 0.94	2.6 ± 1.09
Kumar et al. 2014 [55]	Baseline	-	-	2.90 ± 0.73	3.20 ± 0.78	-	-
	1 w	-	-	0.00 ± 0.00	0.10 ± 0.31	-	-
	3 w	-	-	0.30 ± 0.48	0.30 ± 0.67	-	-
	6 w	-	-	0.50 ± 0.52	0.70 ± 0.82	-	-
	3 mo	-	-	0.90 ± 0.73	0.90 ± 0.87	-	-
	6 mo	61.67 ± 30.22%	68.33 ± 28%	1.00 ± 0.66	1.10 ± 0.99	1.9 ± 0.73	2.1 ± 0.99

C—control group; IQR—interquartile range; M—median; mo—months; RD—recession depth; RecRed—reduction of the recession depth; SD—standard deviation; T—test group; w—weeks. * statistically significant difference between C and T groups.

**Table 4:** Summary of risk of bias of included RCTs (ROB 2).

Author Year	Domains					
	Adequate Sequence Generation?	Allocation Concealment?	Blinding?	Incomplete Outcome Data Addressed?	Free of Selective Reporting?	Free of Other Bias?
Pilloni et al. 2019 [41]	Yes	Yes	Yes	Yes	Yes	No
Rajan et al. 2015 [54]	Unclear	Unclear	Unclear	Yes	Unclear	Unclear
Kumar et al. 2014 [55]	Yes	Unclear	Unclear	Unclear	Yes	Unclear

## Data Availability

The datasets used and/or analyzed during the current study are available from the corresponding author on reasonable request.

## References

[1] Pini Prato G. (1999). Mucogingival deformities. Ann. Periodontol..

[2] Cortellini P., Bissada N.F. (2018). Mucogingival conditions in the natural dentition: Narrative review, case definitions, and diagnostic considerations. J. Periodontol..

[3] Kassab M.M., Cohen R.E. (2003). The etiology and prevalence of gingival recession. J. Am. Dent. Assoc..

[4] Jepsen K., Stefanini M., Sanz M., Zucchelli G., Jepsen S. (2017). Long-Term Stability of Root Coverage by Coronally Advanced Flap Procedures. J. Periodontol..

[5] Joss-Vassalli I., Grebenstein C., Topouzelis N., Sculean A., Katsaros C. (2010). Orthodontic therapy and gingival recession: A systematic review. Orthod. Craniofac. Res..

[6] Merijohn G.K. (2016). Management and prevention of gingival recession. Periodontology 2000.

[7] Khocht A., Simon G., Person P., Denepitiya J.L. (1993). Gingival recession in relation to history of hard toothbrush use. J. Periodontol..

[8] Serino G., Wennström J.L., Lindhe J., Eneroth L. (1994). The prevalence and distribution of gingival recession in subjects with a high standard of oral hygiene. J. Clin. Periodontol..

[9] Rajapakse P.S., McCracken G.I., Gwynnett E., Steen N.D., Guentsch A., Heasman P.A. (2007). Does tooth brushing influence the development and progression of non-inflammatory gingival recession? A systematic review. J. Clin. Periodontol..

[10] Wennström J.L. (1996). Mucogingival therapy. Ann. Periodontol..

[11] Clauser C., Nieri M., Franceschi D., Pagliaro U., Pini-Prato G. (2003). Evidence-based mucogingival therapy. Part 2: Ordinary and individual patient data meta-analyses of surgical treatment of recession using complete root coverage as the outcome variable. J. Periodontol..

[12] Chambrone L., Tatakis D.N. (2015). Periodontal soft tissue root coverage procedures: A systematic review from the AAP Regeneration Workshop. J Periodontol..

[13] Zucchelli G., Mounssif I. (2015). Periodontal plastic surgery. Periodontol 2000.

[14] Grupe H., Warren R. (1956). Repair of gingival defects by a sliding flap operation. J. Periodontol..

[15] Cohen D.W., Ross S.E. (1968). The double papillae repositioned flap in periodontal therapy. J. Periodontol..

[16] Pennel B.M., Higgason J.D., Towner J.D., King K.O., Fritz B.D., Salder J.F. (1965). Oblique Rotated Flap. J. Periodontol..

[17] Bernimoulin J.P., Lüscher B., Mühlemann H.R. (1975). Coronally repositioned periodontal flap. Clinical evaluation after one year. J Clin. Periodontol..

[18] Tarnow D.P. (1986). Semilunar coronally repositioned flap. J. Clin. Periodontol..

[19] Allen A.L. (1994). Use of the supraperiosteal envelope in soft tissue grafting for root coverage. I. Rationale and technique. Int. J. Periodontics Restor. Dent..

[20] de Sanctis M., Zucchelli G. (2007). Coronally advanced flap: A modified surgical approach for isolated recession-type defects: Three-year results. J. Clin. Periodontol..

[21] Zucchelli G., De Sanctis M. (2000). Treatment of multiple recession-type defects in patients with esthetic demands. J. Periodontol..

[22] Aroca S., Molnár B., Windisch P., Gera I., Salvi G.E., Nikolidakis D., Sculean A. (2013). Treatment of multiple adjacent Miller class I and II gingival recessions with a Modified Coronally Advanced Tunnel (MCAT) technique and a collagen matrix or palatal connective tissue graft: A randomized, controlled clinical trial. J. Clin. Periodontol..

[23] Sculean A., Allen E.P. (2018). The Laterally Closed Tunnel for the Treatment of Deep Isolated Mandibular Recessions: Surgical Technique and a Report of 24 Cases. Int. J. Periodontics Restor. Dent..

[24] Carranza N., Pontarolo C., Rojas M.A. (2019). Laterally Stretched Flap With Connective Tissue Graft to Treat Single Narrow Deep Recession Defects on Lower Incisors. Clin. Adv. Periodontics.

[25] Pini Prato G., Clauser C., Cortellini P., Tinti C., Vincenzi G., Pagliaro U. (1996). Guided tissue regeneration versus mucogingival surgery in the treatment of human buccal recessions. A 4-year follow-up study. J. Periodontol..

[26] Pilloni A., Paolantonio M., Camargo P.M. (2006). Root coverage with a coronally positioned flap used in combination with enamel matrix derivative: 18-month clinical evaluation. J. Periodontol..

[27] Zucchelli G., Tavelli L., McGuire M.K., Rasperini G., Feinberg S.E., Wang H., Giannobile W.V. (2020). Autogenous soft tissue grafting for periodontal and peri-implant plastic surgical reconstruction. J. Periodontol..

[28] da Silva R.C., Joly J.C., de Lima A.F., Tatakis D.N. (2004). Root coverage using the coronally positioned flap with or without a subepithelial connective tissue graft. J. Periodontol..

[29] Novaes A.B., Palioto D.B. (2019). Experimental and clinical studies on plastic periodontal procedures. Periodontology 2000.

[30] Tavelli L., Asa’ad F., Acunzo R., Pagni G., Consonni D., Rasperini G. (2018). Minimizing Patient Morbidity Following Palatal Gingival Harvesting: A Randomized Controlled Clinical Study. Int. J. Periodontics Restor. Dent..

[31] Jhaveri H.M., Chavan M.S., Tomar G.B., Deshmukh V.L., Wani M.R., Miller P.D. (2010). Acellular dermal matrix seeded with autologous gingival fibroblasts for the treatment of gingival recession: A proof-of-concept study. J. Periodontol..

[32] Cheng G.-L., Fu E., Tu Y.-K., Shen E.-C., Chiu H.-C., Huang R.-Y., Yuh D.-Y., Chiang C.-Y. (2015). Root coverage by coronally advanced flap with connective tissue graft and/or enamel matrix derivative: A meta-analysis. J. Periodontal Res..

[33] Dahiya P., Kamal R. (2013). Hyaluronic Acid: A boon in periodontal therapy. N. Am. J. Med. Sci..

[34] Chen W.Y., Abatangelo G. (1999). Functions of hyaluronan in wound repair. Wound Repair Regen..

[35] Aya K.L., Stern R. (2014). Hyaluronan in wound healing: Rediscovering a major player. Wound Repair Regen..

[36] Asparuhova M.B., Kiryak D., Eliezer M., Mihov D., Sculean A. (2019). Activity of two hyaluronan preparations on primary human oral fibroblasts. J. Periodontal Res..

[37] Jentsch H., Pomowski R., Kundt G., Göcke R. (2003). Treatment of gingivitis with hyaluronan. J. Clin. Periodontol..

[38] Eick S., Renatus A., Heinicke M., Pfister W., Stratul S.I., Jentsch H. (2013). Hyaluronic Acid as an adjunct after scaling and root planing: A prospective randomized clinical trial. J. Periodontol..

[39] Pilloni A., Zeza B., Kuis D., Vrazic D., Domic T., Olszewska-Czyz I., Popova C., Kotsilkov K., Firkova E., Dermendzieva Y. (2021). Treatment of Residual Periodontal Pockets Using a Hyaluronic Acid-Based Gel: A 12 Month Multicenter Randomized Triple-Blinded Clinical Trial. Antibiotics.

[40] Pilloni A., Schmidlin P.R., Sahrmann P., Sculean A., Rojas M.A. (2019). Effectiveness of adjunctive hyaluronic acid application in coronally advanced flap in Miller class I single gingival recession sites: A randomized controlled clinical trial. Clin. Oral Investig..

[41] Pilloni A., Nardo F., Rojas M.A. (2019). Surgical Treatment of a Cemental Tear-Associated Bony Defect Using Hyaluronic Acid and a Resorbable Collagen Membrane: A 2-Year Follow-Up. Clin. Adv. Periodontics.

[42] Pilloni A., Rojas M.A., Marini L., Russo P., Shirakata Y., Sculean A., Iacono R. (2021). Healing of intrabony defects following regenerative surgery by means of single-flap approach in conjunction with either hyaluronic acid or an enamel matrix derivative: A 24-month randomized controlled clinical trial. Clin. Oral Investig..

[43] Romeo U., Libotte F., Palaia G., Galanakis A., Gaimari G., Tenore G., Del Vecchio A., Polimeni A. (2014). Oral soft tissue wound healing after laser surgery with or without a pool of amino acids and sodium hyaluronate: A randomized clinical study. Photomed. Laser Surg..

[44] Moher D., Liberati A., Tetzlaff J., Altman D.G., PRISMA Group (2009). Preferred reporting items for systematic reviews and meta-analyses: The PRISMA statement. PLoS Med..

[45] Miller P.D. (1985). A classification of marginal tissue recession. Int. J. Periodontics Restor. Dent..

[46] Cairo F., Nieri M., Cincinelli S., Mervelt J., Pagliaro U. (2011). The interproximal clinical attachment level to classify gingival recessions and predict root coverage outcomes: An explorative and reliability study. J. Clin. Periodontol..

[47] Schardt C., Adams M.B., Owens T., Keitz S., Fontelo P. (2007). Utilization of the PICO framework to improve searching PubMed for clinical questions. BMC Med. Inform. Decis. Mak..

[48] Khurshid Z., Tariq R., Asiri F.Y., Abid K., Zafar M.S. (2021). Literature search strategies in dental education and research. J. Taibah Univ. Med. Sci..

[49] Greenhalgh T., Peacock R. (2005). Effectiveness and efficiency of search methods in systematic reviews of complex evidence: Audit of primary sources. BMJ.

[50] Higgins J., Green S. (2011). Cochrane Handbook for Systematic Reviews of Interventions. Version 5.1.0. https://handbook-5-1.cochrane.org.

[51] Higgins J.P.T., Altman D.G., Gøtzsche P.C., Jüni P., Moher D., Oxman A.D., Savović J., Schulz K.F., Weeks L., Sterne J.A.C. (2011). The Cochrane Collaboration’s tool for assessing risk of bias in randomised trials. BMJ.

[52] Brignardello-Petersen R. (2019). Hyaluronic acid used as an adjunct to coronally advanced flap probably results in an increase in recession reduction and root coverage in patients with single Miller class I gingival recessions. J. Am. Dent. Assoc..

[53] Zukorlic H., Jakoba N.N. (2011). Effects of topical application of hyaluronic acid gel on wound healing following gingival recession treatment. Eur. J. Med. Res..

[54] Rajan P., Rao N.M., Nera M., Rahaman S.M. (2015). Hyaluronon as an adjunct to coronally advanced flap for the treatment of gingival recession defects. Natl. J. Integr. Res. Med..

[55] Kumar R., Srinivas M., Pai J., Suragimath G., Prasad K., Polepalle T. (2014). Efficacy of hyaluronic acid (hyaluronan) in root coverage procedures as an adjunct to coronally advanced flap in Millers Class I recession: A clinical study. J. Indian Soc. Periodontol..

[56] Zucchelli G., Stefanini M., Ganz S., Mazzotti C., Mounssif I., Marzadori M. (2016). Coronally Advanced Flap with Different Designs in the Treatment of Gingival Recession: A Comparative Controlled Randomized Clinical Trial. Int. J. Periodontics Restor. Dent..

[57] Dubey P., Narasimhan M., Sehgal N.K., Yanni P., Kim J.W., Kapila Y.L., Lin G.-H. (2021). Connective Tissue Graft with or without Enamel Matrix Derivative for Treating Gingival Recession Defects: A Systematic Review and Meta-Analysis. J. Evid. Based Dent. Pract..

[58] Li F., Yu F., Xu X., Li C., Huang D., Zhou X., Ye L., Zheng L. (2017). Evaluation of Recombinant Human FGF-2 and PDGF-BB in Periodontal Regeneration: A Systematic Review and Meta-Analysis. Sci. Rep..

[59] Miron R.J., Moraschini V., Del Fabbro M., Piattelli A., Fujioka-Kobayashi M., Zhang Y., Saulacic N., Schaller B., Kawase T., Cosgarea R. (2020). Use of platelet-rich fibrin for the treatment of gingival recessions: A systematic review and meta-analysis. Clin. Oral Investig..

[60] Chambrone L., Botelho J., Machado V., Mascarenhas P., Mendes J.J., Avila-Ortiz G. (2022). Does the subepithelial connective tissue graft in conjunction with a coronally advanced flap remain as the gold standard therapy for the treatment of single gingival recession defects? A systematic review and network meta-analysis. J. Periodontol..

[61] Moraschini V., de Almeida D.C.F., Sartoretto S., Bailly Guimarães H., Chaves Cavalcante I., Diuana Calasans-Maia M. (2019). Clinical efficacy of xenogeneic collagen matrix in the treatment of gingival recession: A systematic review and meta-analysis. Acta Odontol. Scand..

[62] Shirakata Y., Imafuji T., Nakamura T., Nakamura T., Kawakami Y., Shinohara Y., Noguchi K., Pilloni A., Sculean A. (2021). Periodontal wound healing/regeneration of two-wall intrabony defects following reconstructive surgery with cross-linked hyaluronic acid-gel with or without a collagen matrix: A preclinical study in dogs. Quintessence Int..

[63] Sasaki T., Watanabe C. (1995). Stimulation of osteoinduction in bone wound healing by high-molecular hyaluronic acid. Bone.

[64] Deed R., Rooney P., Kumar P., Norton J.D., Smith J., Freemont A.J., Kumar S. (1997). Early-response gene signalling is induced by angiogenic oligosaccharides of hyaluronan in endothelial cells. Inhibition by non-angiogenic, high-molecular-weight hyaluronan. Int. J. Cancer.

[65] Bertolami C.N., Messadi D.V. (1994). The role of proteoglycans in hard and soft tissue repair. Crit. Rev. Oral Biol. Med..

[66] Pini Prato G.P., Franceschi D., Cortellini P., Chambrone L. (2018). Long-term evaluation (20 years) of the outcomes of subepithelial connective tissue graft plus coronally advanced flap in the treatment of maxillary single recession-type defects. J. Periodontol..

[67] Gurtner G.C., Werner S., Barrandon Y., Longaker M.T. (2008). Wound repair and regeneration. Nature.

[68] McGuire M.K., Nunn M. (2003). Evaluation of human recession defects treated with coronally advanced flaps and either enamel matrix derivative or connective tissue. Part 1: Comparison of clinical parameters. J. Periodontol..

[69] Snetkov P., Zakharova K., Morozkina S., Olekhnovich R., Uspenskaya M. (2020). Hyaluronic Acid: The Influence of Molecular Weight on Structural, Physical, Physico-Chemical, and Degradable Properties of Biopolymer. Polymers.

